# Effect of botulinum toxin type A on masticatory function and musculoskeletal structure in rabbits

**DOI:** 10.1038/s41598-025-97919-y

**Published:** 2025-05-02

**Authors:** Maha Mohamed Shehata Montaser, Nahed H. Elsokkary, Ahmed Elsayed Abdelghany Ibrahim Shararah

**Affiliations:** 1 Arab Academy for Science, Technology and Maritime Transport- College of Dentistry- Alamein Campus, Alexandria, Egypt; 2 Alexandria University, faculty of Medicine, Azarita, Alexandria, Egypt

**Keywords:** Botulinum toxin type A (BoNT-A), Masseter muscles, Electromyography (EMG), Compound action potential (CAP), Muscle atrophy, Desmin, Cell biology, Health care

## Abstract

Botulinum toxin (BTX) injection could relieve many disorders in the maxillofacial regions. Although it could have some side effects on bones and muscles. This study investigated the effects of a single Botulinum Toxin Type A (BoNT-A) injection on masticatory function and musculoskeletal structure in rabbits. Twenty rabbits were divided into two groups: one received BoNT-A (10 units), and the other received saline. The study periodically monitored electromyography (EMG), compound action potential (CAP), food intake, and body weight, along with histological and immunohistochemical analyses after four weeks. Revealed significant reductions in body weight, EMG, and CAP in the BoNT-A group during the first two weeks, but no notable differences in the following two weeks. Histological examination showed thinning of the alveolar bone trabecula, while ultrastructural changes in the masseter muscle included swollen mitochondria, disorganized Z bands, and heterochromatic nuclei. Light microscopy revealed increased fibrous tissue, muscle fiber breakdown, and vacuolations. Desmin expression was significantly reduced in the BoNT-A group. The findings indicate that a single BoNT-A injection temporarily reduces masticatory function and causes degenerative changes in muscle tissue and bone structure, including alveolar bone resorption, lasting at least four weeks.

**Supplementary Information:**

The online version contains supplementary material available at 10.1038/s41598-025-97919-y.

## Introduction

Botulinum toxin(BTX) is one of the most potent biological poisons, produced by clostridium botulinum, which is present in water, soil, and the intestines of animals^1^ Botulinum toxin injection is used to treat spasms of skeletal muscles, however, in the skull region, it is used to treat hypertonicity of the masseter and temporal muscle to alleviate myofascial pain^2^. Moreover, it is used in curing abnormalities in the head and neck region such as bruxism and dislocation of the temporomandibular joint (TMJ)^3,4^.

Temporomandibular joint disorders can result from muscular dysfunction in the maxillofacial regions^5^, postoperative relapse after corrective jaw surgery^6^, and open bite after maxillofacial trauma^7,8^. This disorder could be relieved by Botulinum toxin (BTX) injection. Some parafunctional habits result in temporomandibular joint disorders such as bruxism^9^. Recently, treatment modalities for bruxism such as Botulinum toxin (BTX) injection aim to reduce symptoms, and muscular tonicity and preserve temporomandibular joint components^10^.

The U.S. Food and Drug Administration (FDA) considers the administration of Botulinum toxin type A (BTX-A) to be safe^11^. However, there have been reports of some adverse effects on muscles, including systemic weakness^12^.

The masseter muscle is one of the primary muscles of mastication, it is strategically positioned to exert considerable force on the mandible^13,14^. Botox significantly reduces muscle hyperactivity by specifically preventing the release of acetylcholine at the neuromuscular junction^15,16^.

Electromyography (EMG) provides information about muscle function, nerve conduction, and any potential abnormalities^17^. However, there is a scarcity of studies that employ EMG to evaluate the effects of BTX injections on the targeted muscles^18,19^.

Desmin is a type of intermediate filament protein, it plays a crucial role in maintaining the structural integrity of muscle fibers, desmin is involved in signaling pathways related to muscle function and repair. This study was the first to test the effect of BTX injection on the desmin level of the masseter muscle after one month of injection^20^.

The mandible is a functional bone that may be impacted by Botox injections. Some studies have examined the effects of Botox injections on the mandible using radiographs and histological analysis of the condyle, but there is limited evidence of its impact on the mandible molar region^21^. We hypothesize that there is no significant difference between both groups after 4 weeks of injection.

This study aims to investigate the impact of a 10-unit dose of BTX-A on the masseter muscles of rabbits over one month, exploring not only muscle function through EMG, CAP, food intake, and body weight, but also covering the histological changes in both muscle and bone using advanced light and electron microscopy. Additionally, it assessed the desmin levels through immunohistochemical techniques to provide a deeper understanding of BTX-A’s broader effects.

## Results

### Body weight and amount of food intake (Table 1)

Table 1 demonstrates a significant difference in food intake and body weight between Group I and Group II over the 4 weeks, with p-values of 0.0058 and 0.01778, respectively; group I had higher values for both measures. During the first two weeks, the differences in body weight and food intake were highly significant (*p* ≤ 0.001), with mean body weights of 1780.57 ± 68.5 for Group I and 1628.57 ± 45.7 for Group II. The mean food intake was 44.51 ± 1.7 for Group I and 39.90 ± 3 for Group II. In the last week, no significant difference was observed in body weight or food intake between the two groups (*p* = 0.3 and 0.065, respectively); however, the p-value for food intake suggests a possible trend toward significance that may warrant further exploration. The mean body weights of 2110 ± 14 for Group I and 2080 ± 28 for Group II. The mean food intake in the final week was 51.82 ± 0.87 for Group I and 50.61 ± 1.3 for Group II.

**Table 1:** Changes in body weight and food intake after 4 weeks, mean, standard deviation, minimum and maximum measurement values for both groups.

	Body weight		Food intake	
Groups	Saline injected	BTX injected	Saline injected	BTX injected
Mean ± SD (gm)	1901.87 ± 131.165	1805 ± 178.23	47.55 ± 3.28	44.48 ± 4.979
95%CI	(1820.44, 1983.30)	(1694.57, 1915.43)	(45.51, 49.59)	(41.40, 47.56)
P-value	0.01778*		0.0058*	
MIN-MAX	1700–2120	1550–2100	42.5–53	35–52

*BTX* botox injection, *Significant (*p* < 0.05), SD: standard deviation, *gm* grams.

### Muscle weight

On the last day in the fourth week, the masseter muscle treated with BTX was approximately 35% lighter than the saline-injected muscle (*p* = 0.002907). The average weight of the saline injected muscle is 2.52 ± 0.7 while the average weight of the BTX injected muscle is 1.63 ± 0.397.

### EMG/CAP examination

As shown in Table 2, the mean values ± SD of EMG amplitude in the saline-injected group were (5.03 ± 0.275.), (5.1 ± 0.34.), (5.01 ± 0.43.), (4.7 ± 0.42.) and (4.81 ± 0.412.) immediately after saline injection, and at the end of the 1st ,2nd, 3rd, and 4th weeks after saline injection respectively. While the mean values ± SD of EMG amplitude in the BTX injected group were (0.056 ± 0.02.) immediately after the injection, (0.195 ± 0.03) after a week post-injection, (0.32 ± 0.07) after 2 weeks post-injection, (3.67 ± 1.25) after 3 weeks post-injection and finally at the end of the experiment after 4 weeks post-injection, the values were (4.3 ± 0.5).

Mean amplitude values were significantly lower (*P* < 0.001) in the BTX injected group compared to the control group immediately after injection as well as at the end of the 1st and 2nd weeks after BTX injection; while at the end of the third week, there was an increase in the mean values of the BTX injected group but still significantly lower in comparison to the third week of the control group (*p* = 0.022). At the end of the 4th week, no significant difference was observed (*P* = 0.063) in the BTX-injected group compared to the control group; Therefore, this p-value suggests a potential trend toward significance that may warrant further investigation.

**Table 2:** Showing the difference means in the EMG in mV between the saline-injected group and the Botox-injected group before injection, immediately after injection, at the end of the first week, second week, third week, and the fourth week after injection.

EMG (mV)	Saline injected (control group)	BTX injected	*P*-value (T-test)
Before injection (95% CI)	5.12 ± 0.3 (4.934, 5.306)	5.16 ± 0.4 (4.913, 5.407)	0.82061681
Immediately after injection (95% CI)	5.03 ± 0.275 (4.859, 5.201)	0.056 ± 0.02 (0.0437, 0.0683)	*P* < 0.001*
1st week after injection (95% CI)	5.1 ± 0.34 (4.89, 5.31)	0.195 ± 0.03 (0.1764, 0.2136)	*P* < 0.001*
2nd week after injection (95% CI)	5.01 ± 0.43 (4.744, 5.276)	0.32 ± 0.07 (0.277, 0.363)	*P* < 0.001*
3rd week after injection (95% CI)	4.7 ± 0.42 (4.44, 4.96)	3.67 ± 1.25 (2.90, 4.44)	0.0242
4th week after injection (95% CI)	4.81 ± 0.412 (4.55, 5.06)	4.3 ± 0.5 (3.99, 4.61)	0.063

*EMG* electromyography, *BTX* botox injection *significant (*p* < 0.05), *BTX* Botex, the p-value of T-test *=statistically significant.

As shown in Table 3 the mean values ± SD of CAP amplitude in the control group were (19.07 ± 1.04), (19.15 ± 1.16), (19.38 ± 1.32.), (18.48 ± 0.996.), and (19.01 ± 0.72.) immediately after saline injection, and at the end of 1st, 2nd, 3rd, and 4th weeks after saline injection respectively. The mean values ± SD of EMG amplitude in the BTX injected group were (2.46 ± 0.33) immediately after the injection, (3.15 ± 0.37) after a week post-injection, (4.02 ± 0.34) after 2 weeks post-injection, (10.75 ± 3.2.) after 3 weeks post-injection, and finally at the end of the experiment after 4 weeks post-injection, the values were (18.33 ± 0.79). Mean Values were significantly lower (*P* < 0.001) in the BTX injected group compared to the control group immediately after injection as well as at the end of the 1st and 2nd weeks after BTX injection; while at the end of the third week, there was an increase in the mean values but still significantly lower in comparison to the third week of the control group (*p* < 0.001). At the end of the 4th week, there was no statistically significant difference (*P* = 0.0595) between the BTX-injected group and the control group; however, this p-value suggests a potential trend toward significance that warrants further investigation with a larger sample size or refined methodology to confirm or refute the observed effect.

**Table 3:** Showing the difference means in the CAP in mV between the saline-injected group and the Botox-injected group before injection, immediately after injection, at the end of the first week, second week, third week, and the fourth week after injection.

CAP (mV)	Saline injected (control group)	BTX injected	*P*-value (T-test)
Before injection (95% CI)	19.51 ± 1.01 (18.89, 20.13)	19.34 ± 1.104 (18.66, 20.02)	0.72371667
Immediately after injection (95% CI)	19.07 ± 1.04 (18.43, 19.71)	2.46 ± 0.33 (2.26, 2.66)	*P* < 0.001*
1st week after injection (95% CI)	19.15 ± 1.16 (18.43, 19.87)	3.15 ± 0.37 (2.92, 3.38)	*P* < 0.001*
2nd week after injection (95% CI)	19.38 ± 1.32 (18.56, 20.20)	4.02 ± 0.34 (3.81, 4.23)	*P* < 0.001*
3rd week after injection (95% CI)	18.48 ± 0.996 (17.86, 19.10)	10.75 ± 3.2 (8.77, 12.73)	*P* < 0.001*
4th week after injection (95% CI)	19.01 ± 0.72 (18.56, 19.46)	18.33 ± 0.79 (17.84, 18.82)	0.05954169

*CAP* compound action potential, *BTX* Botox, p-value of T-test *=statistically significant.

#### Comparative analysis of the rate of change in compound action potential (CAP) and electromyography (EMG) in saline and botox-injected masseter muscles

To calculate the rate of change in the CAP/EMG (mV) from the beginning to the end of the experiment for both the Saline injected and Botox injected groups, we can use the following formula:

$${\text{Rate}}\,{\text{of}}\,{\text{change = }}\tfrac{{{\text{Final}}\,\,{\text{Value-Initial }}\,{\text{Value}}}}{{{\text{Time}}\,\,\,{\text{Period}}}},$$

Where Initial Value is before the injection (the starting value for each group). Final Value is at the 4th week after injection (the ending value for each group). Time Period is the number of weeks between the beginning and the end of the experiment (in this case, 4 weeks).

**Table 4:** Showing the rate of change in compound action potential (CAP) and electromyography (EMG) for saline and botox-injected groups.

Group	CAP rate of change (mV/week)	EMG rate of change (mV/week)
Saline	-0.125	-0.0775
Botox	-0.2525	-0.215

For CAP (mV), the Saline group the saline-injected group had a decrease in CAP of 0.125 mV per week, while the BTX-injected group experienced a slightly faster decrease of 0.2525 mV per week.The saline-injected group had a decrease in EMG of 0.0775 mV per week, while the BTX-injected group experienced a larger decrease of 0.215 mV per week. Therefore, Botox has a greater impact on the CAP (mV), with a larger rate of change compared to the EMG.(Table 4).

### Histopathological results

#### Changes in the alveolar bone (Fig. 1)

**Fig. 1:**
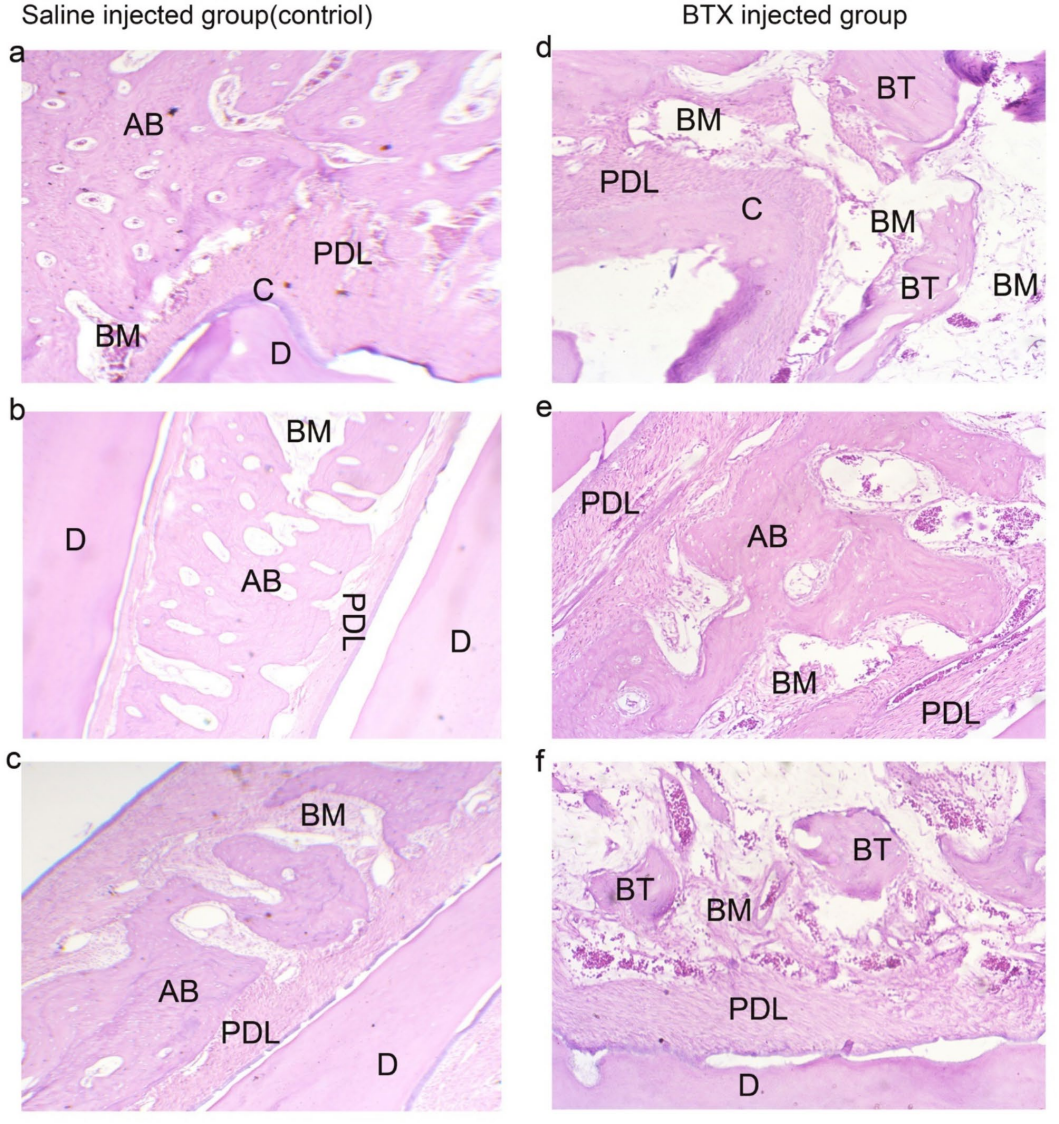
Light micrograph of rabbit’s alveolar bone: (**a**–**c**) after 4 weeks of saline injection and (**d**–**f**) after 4 weeks of botoxulin A injection. (**a**) and (**d**) show changes in the apical bone in both groups with widening in the bone marrow spaces and thinning in bone trabeculae after Botoxulin injection. (**b**) and (**e**) show changes in the interdental bone in both groups with widening in the bone marrow space in the botoxulin A injected group. (**c**) and (**f**) show changes in the alveolar bone present in the middle part of the root with evidence of bone loss in the botoxulin injected group. *AB* alveolar bone, *PDL* periodontal ligament, *C* cementum, *D* dentin, *BM* bone marrow, *BT* bone trabeculae *100.

In the saline-injected group, the apical region featured thick trabeculae surrounding narrow marrow spaces, with well-defined boundaries (Fig. 1a). In contrast, the Botox-injected group displayed cancellous bone with wider marrow spaces and thinner trabeculae (Fig. 1d). The interdental bone between teeth in the saline-injected group (Fig. 1b) had a normal width and density, with bone marrow at varying distances and a high concentration of well-organized osteocytes. However, in the Botox-injected group, the marrow spaces were significantly wider, and the osteocytes were arranged irregularly; with relatively enlarged lacunae (Fig. 1e). In the control group, the alveolar bone along the middle part of the root showed a consistent outline; with a continuous layer of osteoblasts facing the periodontal ligament (Fig. 1c). On the other hand, the Botox-injected group in this region displayed noticeable bone resorption, thinning trabeculae, and an irregular bone surface with discontinuity of osteoblastic cell line (Fig. 1f). Building on previous histological findings, this study reveals that the apical region in the Botox-injected group exhibited the widest bone marrow spaces, the thinnest trabeculae, and a notable presence of osteoclasts along the apical socket walls (supplementary material).

#### Transmission electron micrograph of the masseter muscle (Figs. 2 and 3)

Muscle fibers and cellular structure: in the saline-injected group, sarcomeres were well-aligned and of normal length. In the BoNT-A injected group, a significant distortion with shortened sarcomeres and clear changes in ultrastructure. The arrangement of the fibers remained relatively disorganized and the ultrastructural changes were noticeably different from those observed in the Saline group. (Fig. 3).

**Fig. 2:**
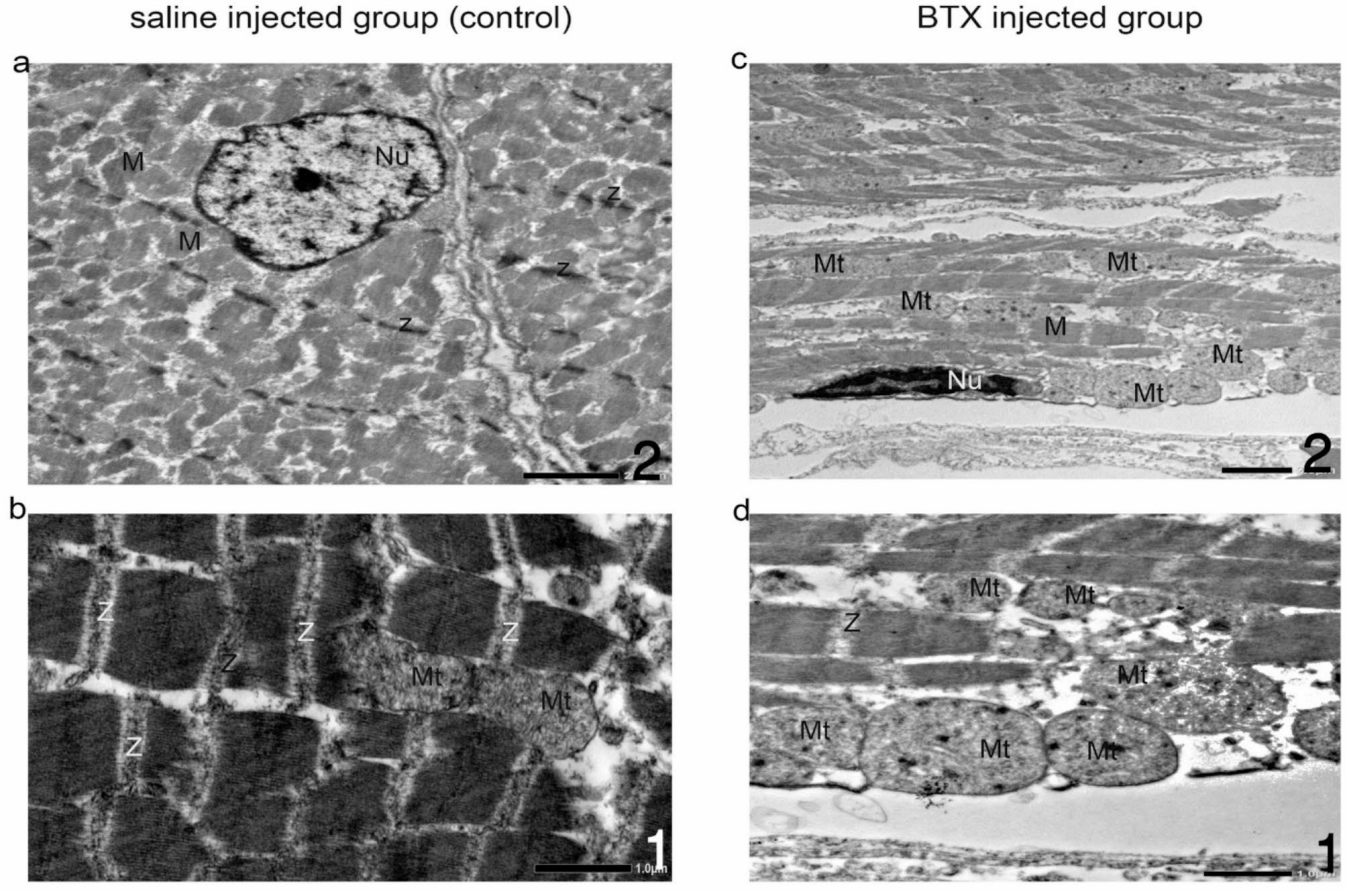
TEM analysis of masseter muscle tissue 4 weeks after saline (**a**,**b**) and botoxulin A injection (**c**,**d**) injection. The saline-injected group (**a**) shows a well-formed euchromatic nucleus (Nu) with a prominent nucleolus and well-structured mitochondria(Mt) with clearly visible cristae (**b**). In contrast, the BTX-A-treated group exhibits shrunken nuclei with chromatin condensation (Nu) (**c**). Swollen mitochondria (Mt) with abnormal, irregular, and degenerated cristae with glycogen deposition (**d**). Magnification: (**a**,**c**) *2000 and (**b**,**d**) *5000. *Nu* nucleus, *M* myofibril, *Z* Z-line, *Mt* mitochondria.

**Fig. 3:**
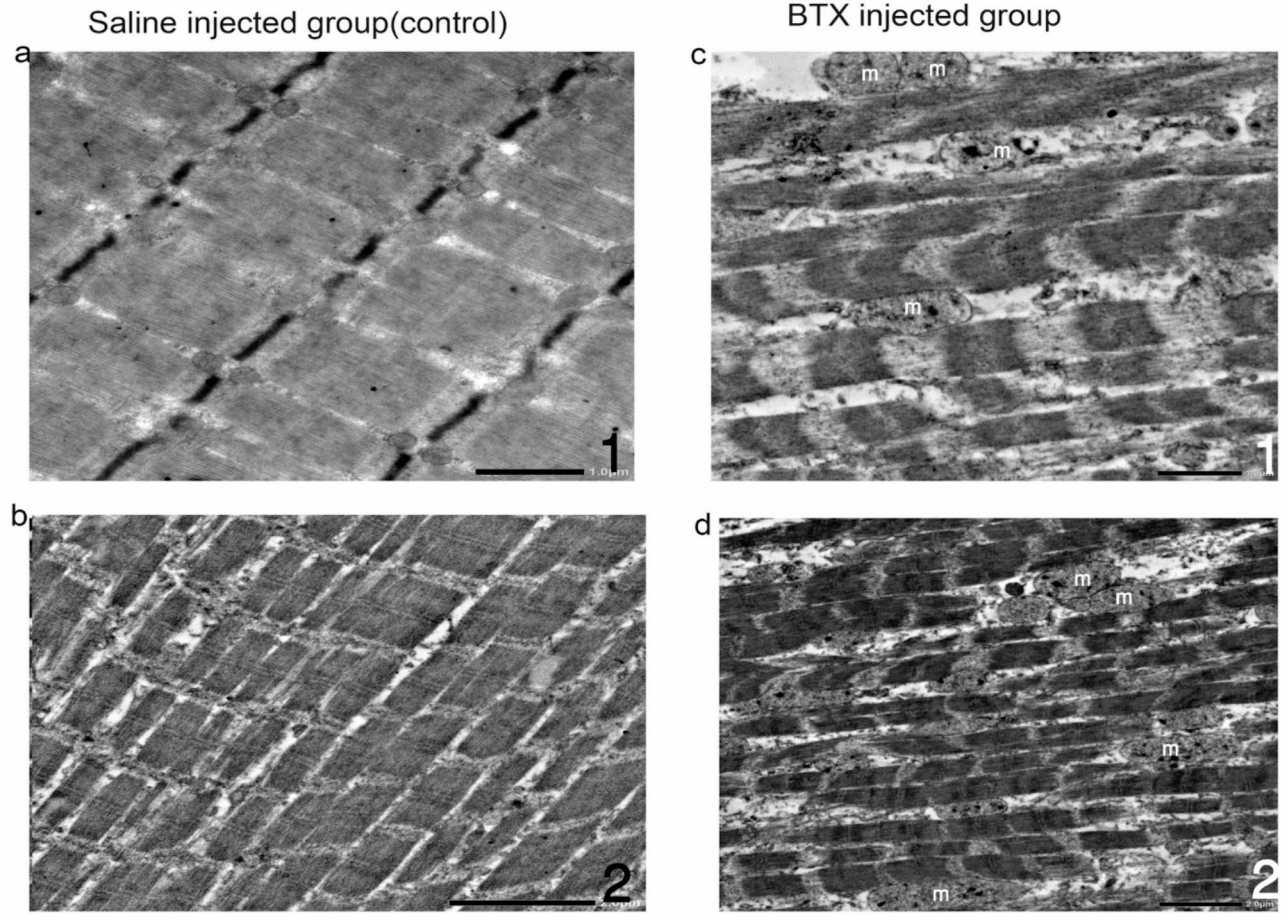
Transmission electron microscopy images displaying the normal structural characteristics of myofibers in animals treated with saline (control group) (**a**,**b**), and disorganization of Z bands and sarcomere shortening in atrophied fibers from animals treated with botulinum toxin type A (**c**,**d**). Magnification: (**a**) *6000, (**b**) *4000, (**c**) *5000, (**d**) *2500. Swollen mitochondria (m), loss of cristae, and glycogen deposition are observed in the samples from the botulinum toxin type A-injected group.

Mitochondria: in the saline-injected group, many mitochondria displayed normal morphology, size, and clear internal structure. (Fig. 2C). In contrast, after BoNT-A injection, normal mitochondria were difficult to observe due to huge vacuolar degeneration and glycogen accumulation. Several mitochondria exhibited myelin sheath-like changes and cristae breakage (Fig. 3D).

Nucleus: in the Botox-injected group, the nuclei appeared dark and heterochromatic, indicating a more condensed chromatin structure. In contrast, the saline-injected group showed euchromatic nuclei, characterized by a more dispersed chromatin structure, suggesting a less condensed state. (Fig. 3a, b)

#### Histological results

**Fig. 4:**
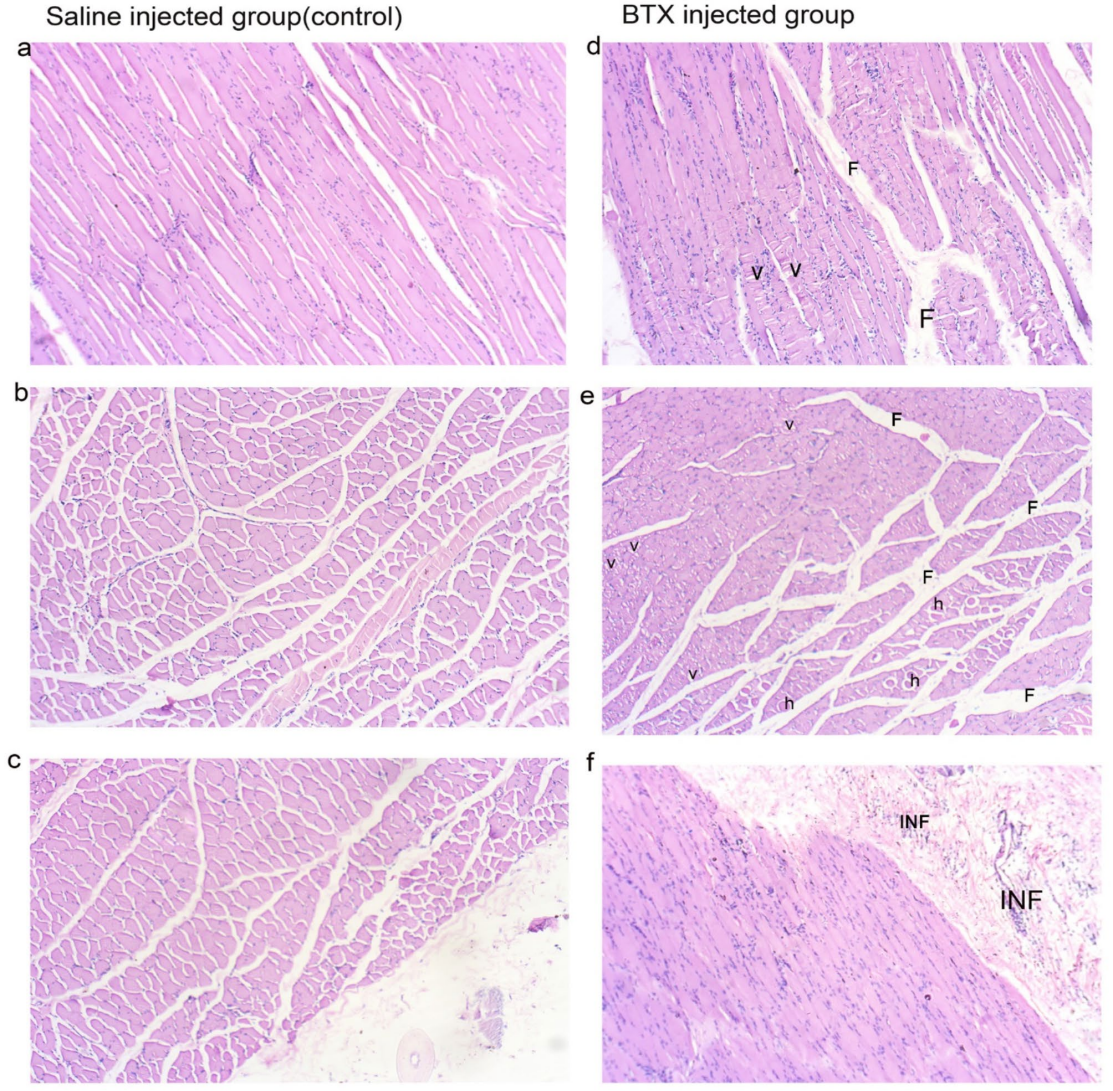
Histological observations showed that the muscle fibers in the saline-injected group (**a**–**c**) exhibited normal myofibril spacing in longitudinal sections (**a**) and a typical polygonal shape in cross sections (**b**). In contrast, the BTX-treated groups (**d**–**f**) displayed irregular and condensed degenerative changes in the muscle fibers in longitudinal section (**d**). The space between myofibers was absent in these groups and degenerated, vacuolated fibers (v) and increased centralized nuclei were present. Some myofibrils exhibited eosinophilic hypertrophy (h) and there was a rise in fibrosis (F) in cross section (**e**), Inflammatory cells (INF) were observed in the connective tissue (**f**). Magnification *100.

**Fig. 5:**
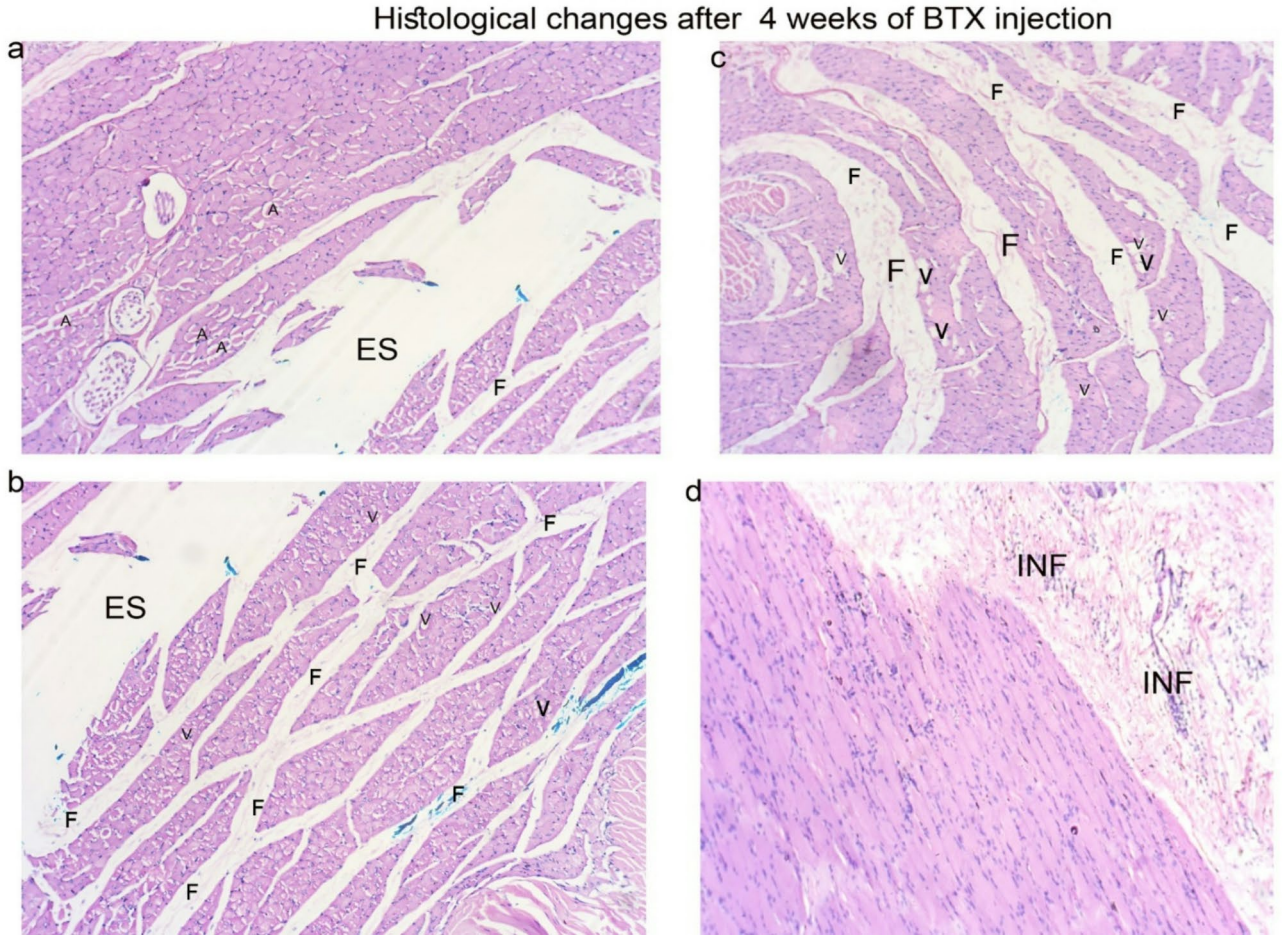
Histological changes in Botox injected group after 4 weeks indicate the inflammatory changes in the masseter muscle, vacuolations (v), fibrosis (F), and inflammatory cells (INF). empty spaces (ES), Atrophic fibers (A) * Magnification *100.

Histologically, degenerative changes in muscle fibers were most prominent in the 10-U BTX-treated group. Fibrous tissue, accompanied by inflammatory cells, was increased and blood vessels were noticeably widened. Additionally, there was a significant rise in the number of nuclei, indicating myofibril regeneration with certain areas showing vacuolations within the muscle fibers. Both atrophy (smaller minimum diameter) and hypertrophy (larger maximum diameter) were observed. Empty profiles suggested either fat accumulation (as adipose tissue dissolves during preparation) or muscle fiber breakdown (Figs. 4 and 5).

#### Morphometric analysis for desmin

**Fig. 6:**
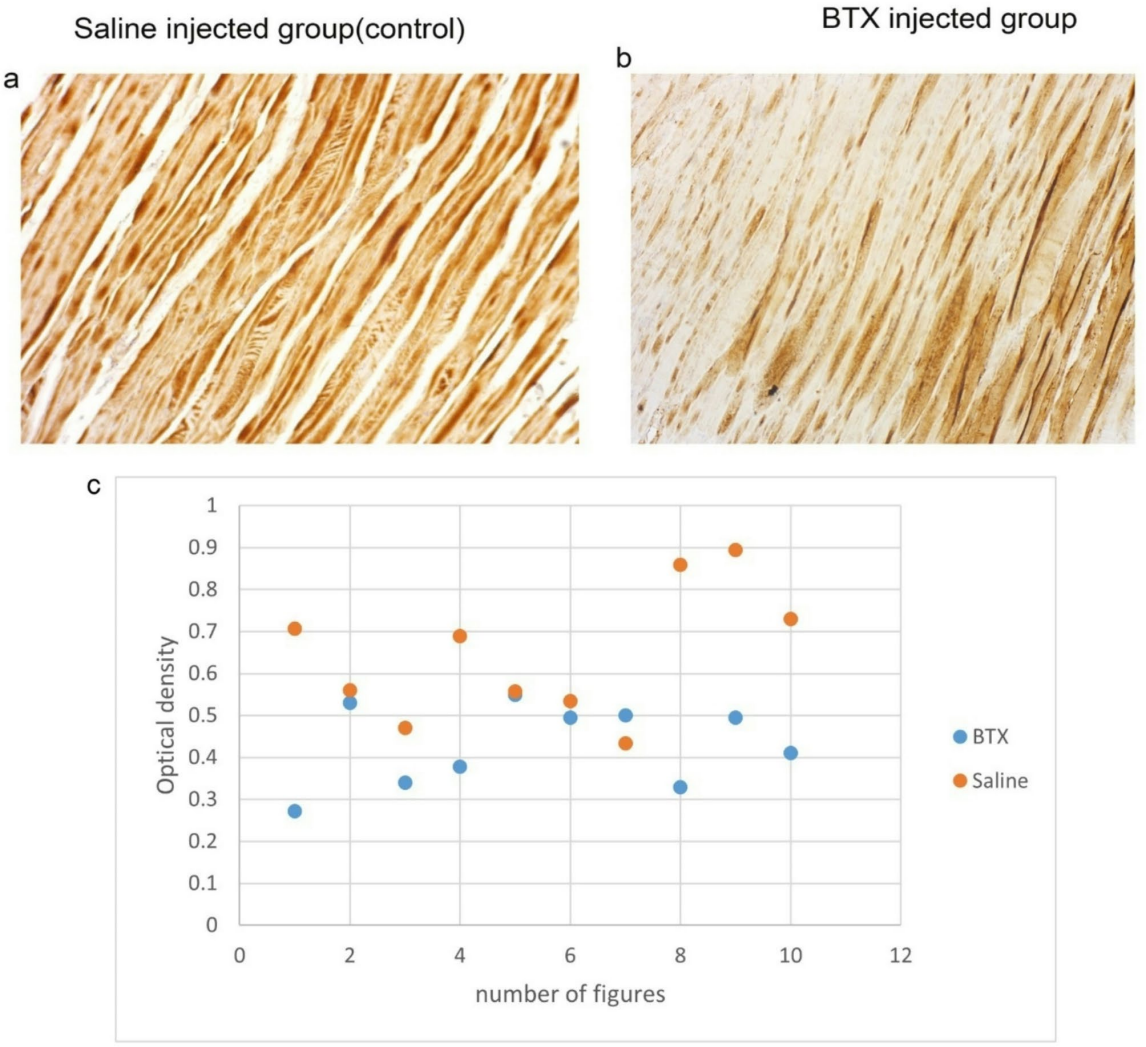
Desmin immunohistochemistry of muscle tissues from the saline-injected and Botox-injected groups is shown, with desmin-positive reactions indicated by brown DAB chromogen in the muscle tissues of both groups. Panels a and b display longitudinal sections, while panel c illustrate the differences between the groups in terms of optical density. The scale bar represents 100 μm.

The saline-treated group exhibited the most intense desmin expression, followed by the Botox-injected group. Additionally, the distribution of desmin in the muscle fibers showed an irregular pattern; with higher concentrations near the edges of the fibers (Fig. 6). Despite these morphological differences, the proportion of desmin-optical density was significantly greater in the saline-injected group compared to the Botox-injected group (*P* = 0.001622) (Fig. 6).

##### Morphometric analysis for mitochondria density

The analysis of the mitochondrial fractional area revealed that the saline group had a mean value of 25.14%, with a standard deviation of 1.63%. The median value for this group was 25%, ranging from 23 to 27.31%. In contrast, the Botulinum toxin (Btx) group had a lower mean mitochondrial fractional area of 13.60%, with a standard deviation of 1.08%. The median value for the Btx group was 13.89%, ranging from 12 to 15%. The statistical analysis showed a highly significant difference between the two groups, with a p-value < 0.05, indicating a significant reduction in mitochondrial fractional area in the Btx group compared to the saline group.

## Discussion

Botulinum toxin (BoNT-A) treatment of the masseter muscle is widely used to treat spasms of skeletal muscles; many studies focus primarily on the histological effects of BoNT in the condylar region, but not on its influence on the alveolar bone, particularly in the molar area. This study aimed to address that gap^22^.

The specific injection sites were selected to be near the motor end plates of the superficial masseter. The 10-unit dosage of BoNT was based on previous rabbit studies and represents the maximum dose that induces changes in muscle and cortical bone thickness. Only female animals were used to minimize the effects of sexual dimorphism^23,24^. All rabbits used in the study were five months old. Although we considered them non-growing adults^25^.

In the current study, there was a significant reduction in muscle weight compared to the saline-injected group. This aligns with findings from Rafferty KL et al., which reported that at the end of the 4th week, the masseter muscle injected with BoNT was on average 18% lighter than the untreated contralateral muscle (*p* < 0.0001)^21^. In this study, after 4 weeks the muscle mass was approximately 35% lower than the saline-injected group. This moderate reduction in muscle mass falls within the 6-40% range reported in studies on rat masseter muscles^26,27^ and mouse hindlimbs^28^, This reduction may be attributed to edema, fibrosis, or fat deposition^29,30^.

Since non-contractile elements like fibrotic or adipose tissue might build up after a BoNT injection, muscle mass alone is not a reliable indicator of muscular force. To give a more direct evaluation of functional ability, we assessed body weight and food consumption. However, real muscle usage is not often reflected by functional capacity alone. Thus, we used electromyography (EMG) to track muscle activity during mastication. This combination of methods, which had never been used before, provides a rare chance to investigate different facets of muscle action and how it affects bone^22^.

In the present study, the BoNT-injected group showed a significant decrease in body weight, food intake, and muscle efficiency measured by EMG compared to the saline group during the first two weeks. However, recovery was observed by the third week, and the two groups had no significant difference in body weight or food intake. This is in line with the findings of Moon et al.^24^, who reported that bilateral BoNT injection into the masseter muscle in rats, using the same dose as this study (10 units, 0.25 ml), resulted in a temporary reduction in food intake; with recovery occurring within 10 days post-injection. Similarly, Liu ZJ et al.^31^ found that bilateral BoNT treatment of the rabbit masseter significantly impaired chewing performance, and deficits in bite force, muscle size, and condylar bone density.

The masseter muscle, which is responsible for jaw movement, consists of both fast-twitch (type II) and slow-twitch (type I) fibers^32^. Fast-twitch fibers are more involved in quick, forceful contractions, while slow-twitch fibers are engaged in sustained, low-intensity activities. One of the differences between fast and slow fibers may arise from the properties of the nerve fibers innervating them. The nerves supplying the “slow” or ‘fatigue-resistant’ fibers are tonically active (a continuous low-frequency output), and this results in the muscles also showing tone. The fast fibers receive bursts of neural activity, whereas the slow fibers have sustained but lower levels of nerve activity^33^.

From our previous results, in the Botox Group, CAP shows a much higher average rate of change compared to EMG. BoNT-A may have a more pronounced effect on fast-twitch fibers due to their greater reliance on neural input for rapid contraction. This could explain why the muscle function was severely impaired in the first two weeks, as the fast-twitch fibers may have been more affected. Over time, as the toxin’s effects wear off and the muscle recovers, there may be a gradual restoration of function in these fast-twitch fibers, leading to the observed recovery.

On the other hand, slow-twitch fibers, which are more resistant to fatigue and play a role in maintaining muscle tone, might be less impacted by BoNT-A, given their lower reliance on rapid neural signaling. An additional assessment of muscle fiber composition by histological analysis or electromyography with different stimulation protocols (e.g., varying frequencies to selectively recruit different fiber types) could provide more insight into the differential effects of the toxin on the two fiber types^34^.(Table 4).

However, additional research has demonstrated that unilateral injection of 10 units of BoNT did not cause any appreciable changes in muscle efficiency, food intake, or body weight^22,35^. These investigations examined how humans and rabbits can maintain proper mastication despite having one-sided nonfunctional masseter muscle. Usually, patients only have short-term chewing difficulty before quickly returning to their regular activities^17,36^. No alterations in masticatory characteristics, such as feeding efficiency, were seen in rabbits. The enlargement of other masticatory muscles might explain this to overcome the loss of masseter force.

In the histological analysis, there were histological changes observed in the group treated with 10 units of Botox. Four weeks post-injection increased fibrous tissue associated with inflammatory cells and widening of blood vessels. This is because BoNT causes muscle paralysis by blocking acetylcholine release, leading to muscle atrophy and cellular changes, which can also provoke an inflammatory response^22^.

A small number of significant autophagic vacuoles were noted in certain fibers associated with cellular damage. Autophagy represents a distinct mechanism of cellular injury, and this observation showing apoptosis in muscle cells treated with BTX-A^37^. Although many recent and past studies do not indicate any destructive changes in the injected muscle, some researchers show degeneration at the cellular level^24,38^.

Centrally located nuclei and a huge number of nuclei were more commonly observed in BTX-A injected masseters compared to saline-injected muscles. This central positioning of nuclei is linked to muscle fiber regeneration. BoNT induces temporary paralysis by blocking acetylcholine. As the fibers try to regenerate, the nuclei shift from their usual peripheral location to the center, a hallmark of muscle repair. This regeneration process often involves inflammatory mediators^22^. As the muscle eventually regains function, the demand for protein synthesis increases, leading to the addition of new nuclei to support the larger muscle mass^39^.

Some myofibrils show hypertrophy as seen in the cross-section slides. This could be explained as the surrounding muscles may become more active to compensate for the reduced activity in the injected muscle, potentially leading to hypertrophy in those muscles^40^.

Fibrosis is a common consequence of denervation, and cranial muscles may be particularly prone to it due to the nature of neural crest-derived fibroblasts^41^. In BTX-A-treated mouse masseters, fibrosis is thought to be related to inflammation^42^. Fibrosis is also associated with increased angiogenesis and vasodilatation^43^. Muscle fibrosis can develop due to injury or disuse; with short-term fibrosis often resolving as the muscle heals, leaving some stiffness. In the long term, repeated damage or prolonged atrophy can lead to persistent fibrosis, causing lasting stiffness and reduced function. Factors like botulinum toxin injection frequency, muscle type, healing ability, and the injury severity affect fibrosis development and duration^22^.

The presence of empty profiles indicated either fat accumulation (as adipose tissue dissolves during preparation) or muscle fiber dissolution. Botox-induced paralysis leads to muscle protein breakdown. The inability of muscles to contract hampers their repair processes, worsening fiber dissolution. In response to muscle atrophy, the body may compensate by depositing fat in areas where muscle mass has decreased, replacing the lost muscle with fat tissue^22^.

Since the literature contains few studies underlying the ultrastructural effects of BTX treatment^44–46^ only after 2 and 12 weeks, we chose to use electron microscopy as an extra parameter to advance light microscopy findings after 4 weeks to determine whether or not the recovery could have begun at the ultrastructural level.

In this study, the ultrastructural findings corroborate the histological results. We observed a disorganized muscle structure; with shortened sarcomeres, mitochondrial swelling, including loss of cristae. Atrophied fibers showed disorganized Z bands with heterochromatic nuclei.

Capra et al.^44^ and Hassan et al.^45^. injected Botox into the masseters of mice and rats. Their findings included muscle atrophy, disorganization of myofibrils and sarcomeres, and focal glycogen accumulation.

These changes are time-dependent and transient, with nearly normal mitochondrial morphology observed 12 months after the injection. Ma et al.^46^ concluded that the muscular changes observed were primarily connected to atrophy as opposed to BTX-A-induced degeneration. This implies that after chemical denervation’s effects subside, the myofibers are expected to return to normal function.

In this study, the mitochondria show significant swelling, degeneration, and glycogen deposition. Glycogen synthase kinase-3β (GSK3β) is located predominantly in the cytosol of the nuclei and mitochondria, which is selectively further activated by some apoptotic stimuli^47^.

Moon et al.^38^ reported that mitochondrial destruction and subsequent autophagy were evident as early as two weeks post-injection. BTX-A injection triggers significant transcriptional changes within one week, activating genes involved in both repair and atrophy pathways, and affecting mitochondrial biogenesis. This leads to an enlargement of mitochondria to meet increased metabolic demands^48^. The results of the quantitative analysis of mitochondria density show a significant reduction in the BTX-A injected group, which could be consistent with the expected effects of muscle inactivity and atrophy induced by Botulinum toxin. It suggests that the reduced muscle activity, caused by the paralysis, might lead to a reduction in mitochondrial biogenesis as the muscle doesn’t require as much energy^49^.

We found some heterochromatic nuclei, which is indicative of cell death. This result is consistent with the idea that skeletal muscle immobility caused by BTX-A, sets off the unfolded protein response and raises endoplasmic reticulum (ER) stress, which leads to apoptosis and cell death. Moon YM et al.^38^ showed comparable results two weeks after injection.

Botulinum toxin (Botox) injections into the masseter muscle typically cause temporary histological changes that affect muscle function; with recovery timelines depending on dosage and frequency. Muscle function generally begins to recover within 4 weeks; with full recovery occurring within 3–6 months^22,50^. A study by de Souza Nobre et al. (2024) found no significant differences in masticatory performance between saline and Botox (75 IU) groups after six months^50^. Histological recovery is usually complete within 3–6 months^46,51^, Ma et al. (2018) found that most ultrastructural changes in the masseter muscle were temporary and resolved within 12 months after Botox injections^46^.

The immunohistochemical results showed a significant reduction in desmin expression in the Botox-injected group compared to the saline group. This reduction is due to Botox-induced muscle paralysis and atrophy, which disrupt muscle structure. Desmin, which is essential for maintaining muscle fiber stability, becomes disorganized or fragmented during atrophy and degeneration, weakening the muscle’s cytoskeletal integrity.

Meyer GA et al.^52^ demonstrated a connection between fibrosis and increased cellular damage in muscles that do not have the desmin filament network’s cytoskeletal support. In contrast, Zhang Y et al.^53^, in a study on rats with induced occlusal trauma, found that desmin expression decreased in both the traumatic and non-traumatic sides of the masseter in a time-dependent manner, reaching its lowest point by the 14th day and returning to normal by the 28th day. This was unlike our immunohistochemical results which remained decreased after 4 weeks. This was attributed to occlusal trauma, which triggered nerve stimulation in the masseter, activating neurochemicals that sent signals to the central nervous system (CNS). In response, the CNS issued instructions, such as ciliary neurotrophic factor (CNTF) secretion, to resist tissue injury. CNTF activated the JAK/STAT pathway, forming a protein complex that entered the nucleus, initiating transcription and enhancing resistance to damage, this differs from the current study, which used Botox injections. Unlike occlusal trauma, Botox blocks neurotransmitter release, leading to muscle disuse and atrophy rather than nerve stimulation. While occlusal trauma triggers regenerative pathways, such as the activation of CNTF and the JAK/STAT pathway to resist damage, Botox-induced muscle inactivity results in atrophy without such protective mechanisms.

While direct studies on the effects of botulinum toxin (BoNT) on dystrophin, titin, or filamin C are limited, research suggests that BoNT can influence muscle proteins and structure, potentially affecting these cytoskeletal components. A study on mice found that BoNT-A caused prolonged de- and regeneration processes in masticatory muscles; reducing dystrophin expression and increasing caveolin-1 and caveolin-3 levels, which are linked to muscle cell membrane integrity^42^. Another study on rat gastrocnemius muscles showed that BoNT-A altered myosin heavy chain (MyHC) isoform composition, indicating changes in muscle fiber type and function, which could indirectly affect other muscle-related proteins(titin and filamin C)^54^.

Many studies have examined the effects of Botox injections on the mandible using radiographs and histological analysis of the condyle only, but there is limited evidence of its histological impact on the alveolar bone region^21,26–29^. The alveolar process, being a functioning bone, is particularly sensitive to reduced muscle activity caused by Botox injections. This study aims to address this gap. The histological findings in the molar region of the mandible in the Botox-injected group revealed signs of bone resorption, including thinning of trabeculae and increased bone marrow spaces. Additionally, the bone surface showed irregular indentations or cavities where osteoclasts had resorbed the bone matrix. This was observed in the apical region. This phenomenon is explained by Wolff’s Law, which suggests that bones remodel in response to mechanical stress. Reduced activity in the masseter muscle decreases the mechanical load on the mandible, weakening the stimulus for bone formation and leading to increased bone resorption, particularly in areas where forces are typically the highest, such as the apical (tip) regions of the teeth, where bone is often more directly influenced by the forces of muscle contraction^55^ (Supplementary material).

These outcomes are in line with those of Tsai C-Y et al.^26^, who used botulinum neurotoxin type A (BoTx/A) to induce masticatory hypoactivity in developing rats by injecting it into their masseter and temporalis muscles. The rats’ decreased cortical bone thickness and bone mineral density (BMD) in the skull and mandibular bone structure 45 days after injection further supported the association between decreased muscular activity and bone resorption.

After botulinum toxin (BoNT-A) injection into the masseter muscle, long-term alveolar bone recovery involves complex remodeling. The reduced muscle activity from BoNT-A decreases mechanical loading on the alveolar bone, triggering osteoclast activation and bone resorption through the RANKL/OPG signaling pathway. Recovery occurs in two phases: the acute phase (0–6 weeks), where muscle paralysis leads to bone resorption, and the chronic phase (3 months to 1 year), where bone remodeling begins as muscle function recovers. Bone recovery relies on the restoration of chewing forces to stimulate osteoblasts, but this process is slower than bone resorption^56^.

## Methods

### Ethical statment

An experimental study was conducted from August 1st, 2024, to the end of the month at the animal house of AASTMT. All animal procedures were approved by the Committee for Care and Use of Laboratory Animals at AASTMT Alamein Dental University, with ethical number (131/2024). This study is performed in accordance with relevant guidelines and regulations. All methods are reported in accordance with ARRIVE guidelines., as well as consulting the ​American Veterinary Medical Association​ (AVMA) Guidelines for the Euthanasia of Animals (2020)​, as a comprehensive resource for guidance on veterinary best practices for the anaesthesia and euthanasia of animals^21^.

### Animal housing and allocation^21^

Twenty healthy female New Zealand rabbits, each weighing approximately 2000 g (around 4 months old), were obtained from the animal facility at the Medical Research Institute, Alexandria University. The rabbits were housed individually in a controlled environment with a 12-hour light/dark cycle, a constant temperature of 22 °C, and 50% humidity throughout the 4-week experiment.

The rabbits were randomly divided into two groups of 10 rabbits each using computer-generated randomized numbers:

- Group I (Control group): Rabbits received a saline injection into both masseter muscles.
- Group II (BTX-injected group): Rabbits received a 10-unit Botox (BTX) injection into each masseter muscle on both sides. Each masseter muscle had ten units (0.25 ml) of BTX, dispersed evenly throughout the frontal, center, and posterior portions.

Before injection, anesthesia was administered using isoflurane. The rabbits were acclimatized to the laboratory environment for approximately two weeks before the experiment began. During this acclimatization period, baseline functional assessments were performed by measuring daily food intake and body weight. Surface EMG recordings were conducted for separate days before and after the injection, synchronized with video footage using QuickCam (Logitech, Fremont, CA).

### Sample size calculations

The sample size was determined using Power Analysis and Sample Size Software (PASS 2020) by NCSS, LLC, Kaysville, Utah, USA. The effect size was based on a study by Young-Min Moon et al. (2015), two weeks of Botox (BTX) injections led to a reduction in the force of masseter muscle contractions and a decrease in muscle size. Consequently, there was also a reduction in bony deposition and cortical bone thickness of the ramus compared to the control group of rabbits. A minimum total sample size of 20 eligible subjects (10 per group) was calculated as necessary to evaluate the functional and histological effects of Botox injections into the masseter muscle. The calculation was made considering a 30% assumed effect size (Minimally Clinically Important Difference), a 95% confidence level, and a compliance ratio of 1:1. This calculation also assumed a 25% effect size, a significance level of 5%, and a power of 80%; using the Chi-square test^24,57^.

The sample size was calculated using this formula:


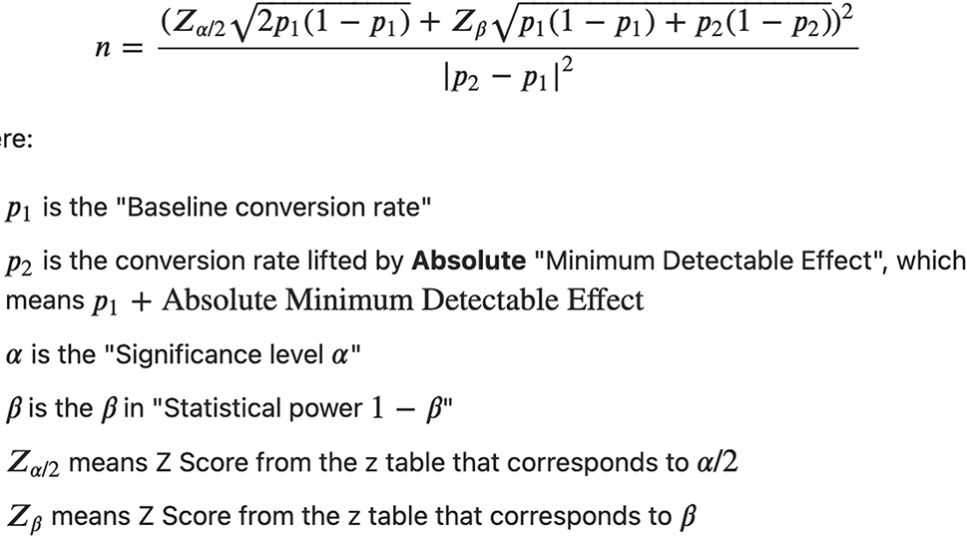


### Diet composition

Food composition: rabbit pellets typically provide 12–14% protein, up to 3% fat, 0.5–0.8% calcium, and at least 18% fiber^58^.

### Functional assessment of masseter muscle

#### Body weight and amount of food intake

Food intake was measured as the amount consumed per rat per day (in grams) by weighting the food in each cage dispenser and accounting for any food spilled on the cage floor. The data were then normalized between the two groups. Throughout the study, the rabbits had unrestricted access to tap water. The body weight of all animals was recorded and monitored daily from day 0 (before the intervention) until the end of the experiment at 4 weeks^21^.

#### EMG examination^59^

On the rabbit’s undamaged, shaven skin, two stimulating electrodes were positioned along the trigeminal nerve root. The rabbit’s masseter muscle was fitted with two recording electrodes. For around ten minutes, the muscle’s baseline electric activity was recorded to capture the muscular EMG. The muscle compound action potential was then recorded using an electric stimulator that produced a monophasic, single, square pulse with a duration of 1 ms and an intensity of 10 mA (EMG100C; Biopac Systems Inc., CA, USA). Until the supramaximal stimulation that guaranteed maximal amplitude was attained (1 mA), the intensity was progressively raised. An MP 150 (Biopac Systems Inc.) was then used to digitally transform the captured signals. The electromyograph’s duration and amplitude were recorded. Throughout the testing, the rabbit’s body temperature was maintained at about 37 °C using a heating light^59^.

#### Muscle weight

Four weeks after the injection, the masseter muscle was carefully extracted from the rabbits after euthanization sacrification to be weighted in grams, and the average weight for each group was calculated^21^.

### Histological assessment of masseter muscle and alveolar bone

The animals were sedated during the fourth week to sample them for light and electron microscopy analyses, and 1 × 1 cm muscle samples were taken.

### Histological procedure for masseter muscle and mandible

All animals were euthanized with an overdose of intraperitoneal sodium pentobarbital anesthesia (100 mg/kg). Fresh biopsy specimens were cut longitudinally into two halves, one for the longitudinal sections and the other for the transverse sections, then the specimens were preserved in 10% neutral-buffered formalin, followed by washing, and the mandibles were decalcified over 2 weeks using formic acid. Following escalating ethanol concentrations, the masseter muscles and mandibles were dehydrated, cleaned with xylene, and then embedded in paraffin wax blocks. After slicing to a 4 μm thickness, sections were stained with hematoxylin and eosin. At the AASTMT biology laboratory, these stained slices were seen under a light microscope for additional examination^24,60^.

### Immunohistochemical of the masseter muscle

For immunostaining, serial portions of the masseter muscle were put on charged positively slides (Superfrost by Fisher Scientific). The slides were placed in a 98 °C heated mixture (WCAP, citrate pH 6; BiOptica, Milan, Italy). for 20 min to expose antigens. Endogenous peroxidase activity was inhibited using Dako REAL Peroxidase-Blocking Solution (S2023, Dako, Glostrup, Denmark), and tissues were treated with 2.5% normal horse serum (ImmPRESS reagent kit, Vector Labs, Burlingame, CA, USA) and 2% bovine serum albumin (BSA) to prevent non-specific binding. After that, the sections were incubated with primary antibodies against desmin (1:200 dilution) for an entire night at 4 °C. The sections were then treated with 3,3’-Diaminobenzidine (DAB) chromogen (ImmPACT DAB; Vector Laboratories) after being treated with an anti-rabbit secondary antibody (ImmPRESS reagent kit—peroxidase—MP-7500; Vector Laboratories, Burlingame, CA, USA) for 30 min at room temperature. The tissues were dried, mounted, and counterstained with hematoxylin. A Nikon Ds-fi1 camera was used to take digital pictures of the slides as they were viewed under a light microscope (Nikon Eclipse 80i)^61^.

### Morphometry for desmin immunohistochemistry^62^

At a ×100 magnification, ten non-overlapping areas were chosen at random from each animal and examined. In addition to an Olympus BX53 microscope, measurements were performed using Image Pro Plus v6.0 (Media Cybernetics, Maryland, USA) and NIH ImageJ (v1.49) (http://rsb.info.nih.gov/ij/). Desmin immunostaining’s optical density (OD) and area % were evaluated. The formula OD = log (max intensity/mean intensity) was used to determine OD; for 8-bit pictures, the maximum intensity is 255.

### Morphometry of mitochondria density

The fractional area of intermyofibrillar mitochondria was assessed using electron microscopy (EM). A total of 20 images (10 per group) were selected from each rabbit, with no visible mechanical damage, and analyzed at ×5,000 magnification. The same observer analyzed all images. The digitized images were processed in ImageJ software (version 1.38X) to isolate mitochondrial structures using the “Threshold” function and were analyzed for mitochondrial fractional density, which represents the proportion of the fiber area containing mitochondria. Mitochondrial density (mitochondria per unit area) was quantified to enable comparisons between the saline-injected and Botulinum toxin (Btx) injected groups^49^.

### Transmission electron microscope examination of masseter muscle

A 100 mg/kg intraperitoneal sodium pentobarbital anesthetic overdose was used to end the lives of all the animals that were slaughtered. The biopsies were immediately fixed in 2.5% (w/v) glutaraldehyde, post-fixed with osmium tetroxide, and embedded in Epon-812 for examination by light and electron microscopy. The new biopsy specimens were cut longitudinally in half, one half for the longitudinal sections and the other half for the transverse sections. Sections that were one micrometer thick were stained with toluidine blue and seen under a light microscope. An ultramicrotome (Leica, Germany) was used to create thin slices for electron microscopy. These sections were then placed on copper grids, coated with uranyl acetate, and examined on a Jeol 1010-B electron microscope^48^.

### Data management and statistical analysis

The results for body weight, muscle weight, food intake, and desmin antibody expression were presented as the mean, maximum, minimum, and standard deviation for the 10 rabbits in each group. Comparisons between the control and Botox injected groups were made using the independent samples t-test to determine if the group means were statistically equal. The Kruskal-Wallis ANOVA was employed to examine variations in the timing amplitude of EMG activity. A p-value below 0.05 was considered statistically significant. Statistical analyses were carried out using SPSS version 23.0 for Windows at the Medical Research Institute, University of Alexandria^21,61^.

## Conclusion

Based on the findings of the study, there was a significant reduction in the desmin level indicating muscle damage or dysfunction. This reduction in desmin suggests that the muscle’s regenerative capacity may be insufficient to fully restore its structure and function if the damage is extensive.

Additionally, there was a significant short-term reduction in masticatory function for up to two weeks. More concerning, there were degenerative changes in the masseter muscle at both histological and ultrastructural levels, with a significant reduction in mitochondrial density. Moreover, there was a noticeable loss of alveolar bone in the apical region lasting up to four weeks post-injection.

## Electronic supplementary material

Below is the link to the electronic supplementary material.

Supplementary Material 1

Supplementary Material 2

## Data Availability

Data is provided within the manuscript or supplementary information files.
