# Supplementary figures and images for: Effect of botulinum toxin type A on masticatory function and musculoskeletal structure in rabbits
Source: Sci Rep. 2025 May 2;15:15323. doi: 10.1038/s41598-025-97919-y (PMC12045985; doi:10.1038/s41598-025-97919-y)

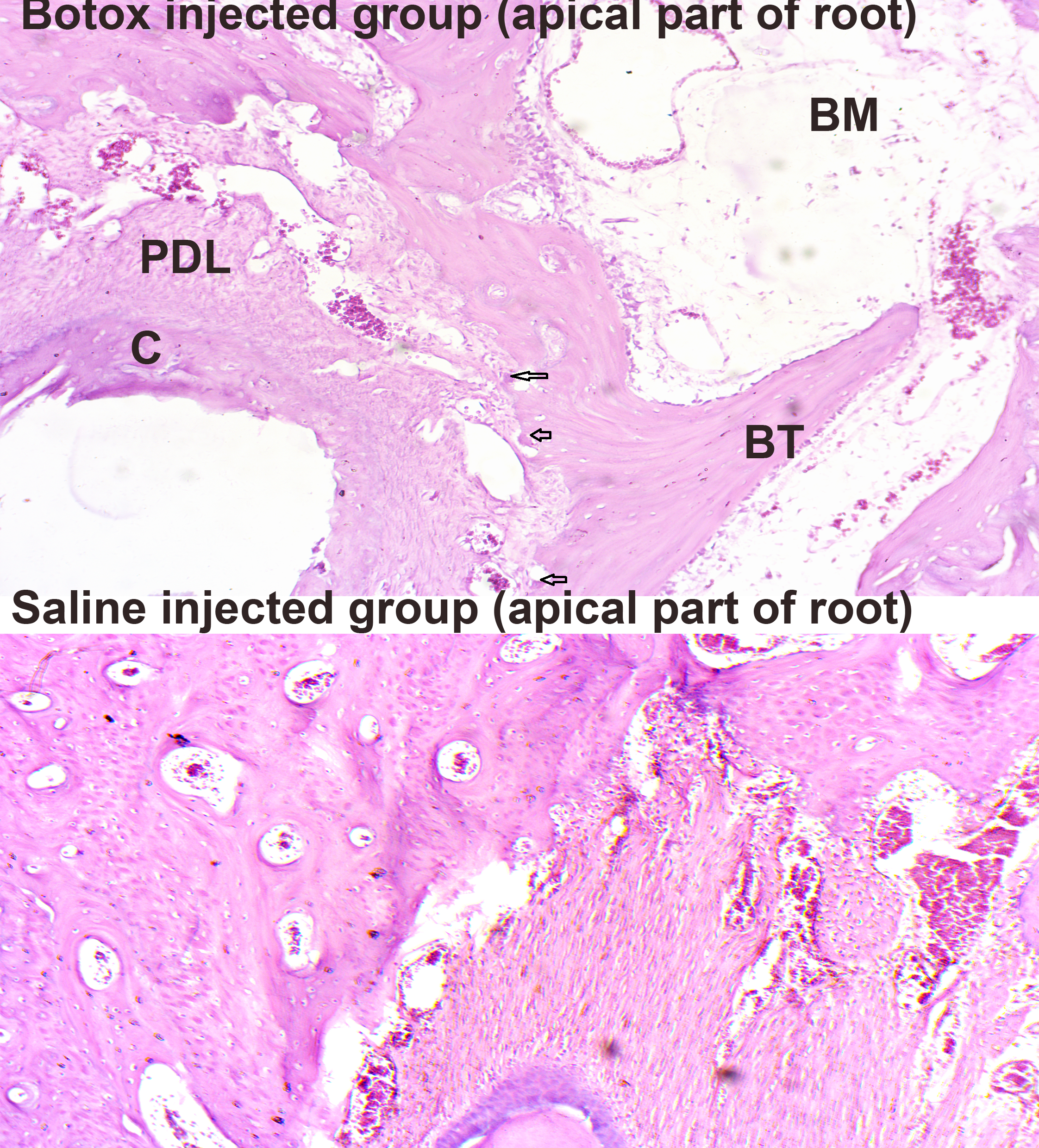

Supplement: Supplementary file 1 — Supplementary Material 1 [file 41598_2025_97919_MOESM1_ESM.tif]
