# Supplementary material for: Effect of botulinum toxin type A on masticatory function and musculoskeletal structure in rabbits
Source: Sci Rep. 2025 May 2;15:15323. doi: 10.1038/s41598-025-97919-y (PMC12045985; doi:10.1038/s41598-025-97919-y)
Supplement: Supplementary file 2 — Supplementary Material 2 [file 41598_2025_97919_MOESM2_ESM.docx]

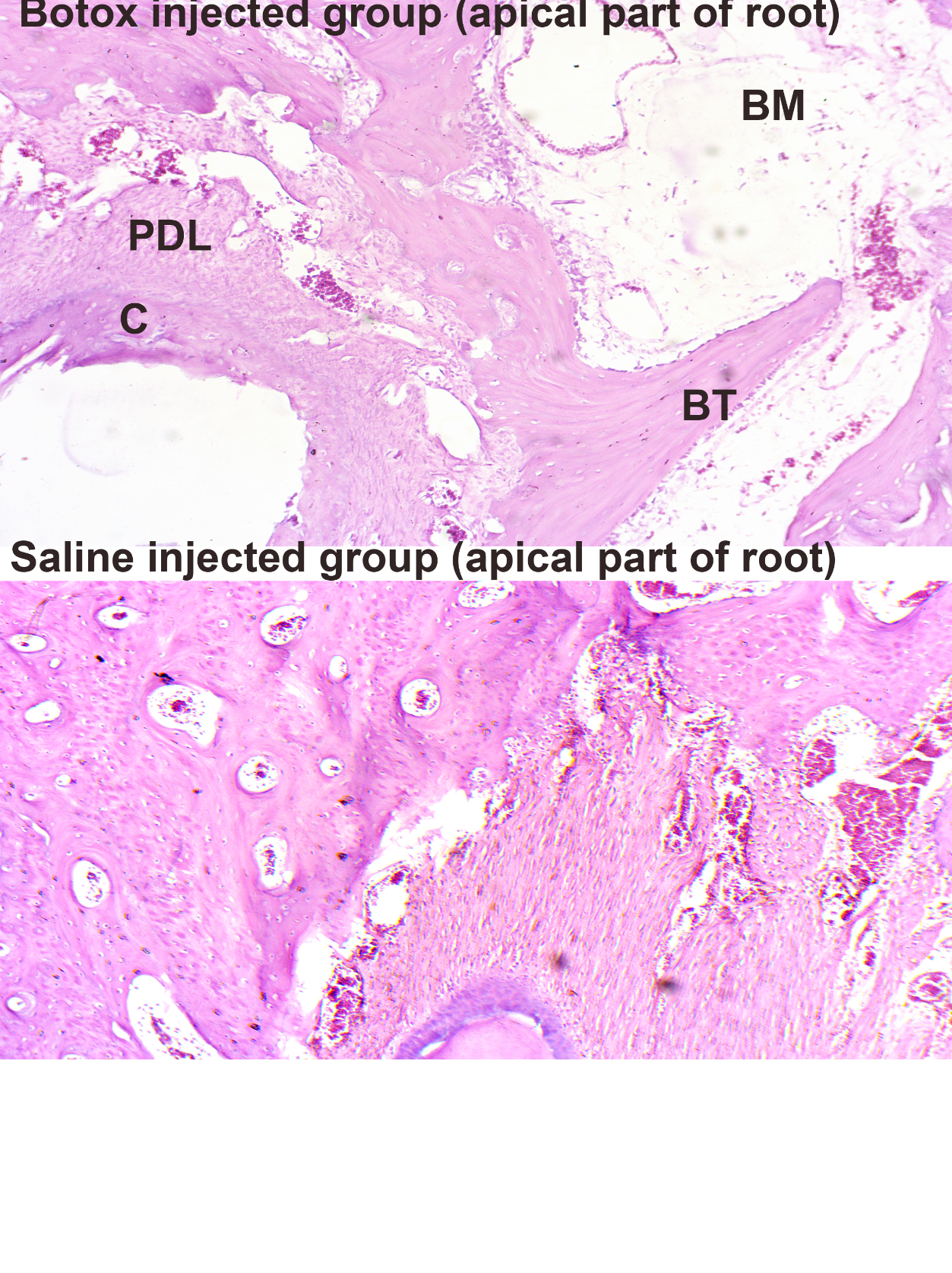


**C**

Light micrograph H & E showing:The upper portion of the figure: Botox injected group of the apical region of the alveolar bone showing irregular border of the base of the socket. Note the marked thinness of the bone trabeculae. Arrows showing osteoclasts at the base of the sockets, disorganized periodontal ligaments. *100 magnification

Lower portion of the figure: The apical portion of the alveolar bone showing normal histological features, with normal density and distribution of the osteocytes, well organized apical periodontal fibers. *400 magnification

**BT**

**BM**

**PDL**
